# An Exercise in Clinical Reasoning: Use of Social Context in Diagnosing an Elevated Lactate

**DOI:** 10.1007/s11606-024-08831-6

**Published:** 2024-09-04

**Authors:** Rachel Si-wen Chang, Gregory M. Ow, Evan J. Walker, Katherine Brooks, Andrew R. Lai

**Affiliations:** 1 Department of Medicine, University of California San Francisco, San Francisco, CA USA; 2 Division of Hematology and Oncology, Department of Medicine, University of California San Francisco, San Francisco, CA USA; 3 Helen Diller Family Comprehensive Cancer Center, University of California San Francisco, San Francisco, CA USA; 4 Division of Hospital Medicine, Department of Medicine, Zuckerberg San Francisco General Hospital, University of California San Francisco, San Francisco, CA USA; 5 Division of Hospital Medicine, Department of Medicine, University of California San Francisco, San Francisco, CA USA

In this series, a clinician extemporaneously discusses the diagnostic approach (regular text) to sequentially presented clinical information (**bold**). Additional commentary on the diagnostic reasoning process (*italic*) is integrated throughout the discussion.

**A 65-year-old man (he/him) presented to a San Francisco hospital for ongoing severe sinus congestion, drainage, and pressure and was admitted for a lactate of 10 mmol/L (0.5–2.2 mmol/L). Medical history was notable for gastric cancer (diagnosed 15 years ago, treated with partial resection and chemotherapy) and nasal polyps with recurrent sinusitis (diagnosed 6 months ago, repeatedly treated with antibiotics). He did not take metformin or albuterol. He lived with his wife and previously worked as an electrician. He formerly smoked (25 pack-years), occasionally drank alcohol, and did not use other drugs. He was born in the city of Guangzhou in the province of Guangdong, China, and immigrated to the United States (U.S.) in 2009 (13 years prior to presentation). His primary spoken language was Cantonese.**

The patient is presenting with chronic rhinosinusitis, a syndrome including nasal congestion and drainage, facial pressure, and headache. Common etiologies include allergic or infectious causes, medications, and environmental exposures. This patient’s syndrome fits the label of chronic rhinosinusitis with nasal polyps. The lack of response to antibiotics makes bacterial infection less likely. While this is his presenting concern, the markedly elevated lactate is striking and warrants concurrent discussion—especially assuming the presence of an associated acidosis.

Lactic acidosis can be divided into type A (related to tissue hypoperfusion or ischemia) and type B (unrelated to tissue hypoperfusion or ischemia). Type A can be caused by systemic shock or local hypoperfusion, such as mesenteric or limb ischemia, and should be rapidly evaluated with vital signs and physical exam. If there is no evidence of these, causes of type B lactic acidosis (including liver disease, cancer, medications, thiamine deficiency, alcohol ingestion, human immunodeficiency virus [HIV], congenital causes) should be explored. A lactate of 10 is quite high; among etiologies of type B lactic acidosis, it is more commonly seen in cancer or thiamine deficiency.

Notably, the social history is specific, including his country, birth region, and migration history. This helps us understand the duration of his life spent in China and in the U.S., informing his risk of possible exposures. Without these details, we might broadly categorize him as “Asian” and “immigrant,” which are more likely to trigger potential unintended or unconscious biases that could negatively influence clinical reasoning. Language preference should also be noted early—to document the need for a professional interpreter and center clinician awareness that diagnostic errors may be more likely when patients cannot communicate directly in their primary language.^1^

I would summarize his problem representation as: 65-year-old man with remote history of gastric cancer presenting with antibiotic-refractory chronic rhinosinusitis, found to have significant lactic acidosis.

*Clinical reasoning requires integration of clinical findings to generate a problem representation (PR), a one-sentence summary highlighting the defining case features in abstract terms.*^2,3^ *Summarizing patient-specific information into abstract semantic qualifiers (acute vs chronic) adds differentiating power. The discussant has elected to center his PR around the most striking feature: the lactic acidosis. Notably, while he acknowledges the specific social history, he does not utilize it in his PR or approach to lactic acidosis. In contrast to the historical practice of including race in one-line assessments, current consensus is to avoid the routine use of race in diagnostic framing to avoid the false implication that it can serve as a valid proxy for genetic or biologic risk.*^4^

**His initial temperature was 36.7 °C, blood pressure 109/58 mmHg, heart rate 93 beats per minute, and oxygen saturation 99% on ambient air. He was well-appearing, thin, and ambulatory, and had normal cardiac, pulmonary, and abdominal exams. Neurologic exam was normal except for decreased left hearing acuity. He had no cervical or supraclavicular lymphadenopathy. He developed mild epistaxis, which resolved with nasal packing. Further history was notable for drenching night sweats, a 10-pound weight loss in 3 months (7% of body weight), occasional orthostasis, ambulatory vertigo, left-sided tinnitus, and intermittent epistaxis. He reported no fevers, chills, hemoptysis, cough, dyspnea, nausea, vomiting, melena, hematochezia, hematuria, or other bleeding/bruising.**

The constellation of symptoms, history, vital signs, and exam findings do not indicate systemic hypoperfusion or local ischemia. Therefore, this presentation is most consistent with type B lactic acidosis. His B symptoms raise concern for subacute to chronic infection, malignancy, or another inflammatory process. Hyperviscosity syndrome as a malignant complication should also be considered given the headaches, epistaxis, and hearing loss.

The chief concern of rhinosinusitis, along with unilateral tinnitus, vertigo, and epistaxis, all localize to the head. Therefore, testing should include magnetic resonance imaging (MRI) to evaluate the sinuses, posterior cerebral fossa, and internal auditory canal.

Here is my updated PR: 65-year-old man with remote history of gastric cancer presenting with antibiotic-refractory chronic rhinosinusitis, co-localizing unilateral hearing loss and epistaxis, and subacute B symptoms, found to have type B lactic acidosis.

*PRs require synthesizing disparate signs and symptoms into higher-level abstractions called syndromes. The syndrome of “co-localizing symptoms” provides anatomic direction for further workup. Weight loss and night sweats have been merged into the syndrome of B symptoms. The discussant has not yet utilized the patient’s country of origin or migration history, which is appropriate to avoid anchoring while the differential remains broad.*

**Laboratory evaluation demonstrated a white blood cell count of 4200/mm** ^**3**^ **(differential notable for mild basophilia and rare blasts), hemoglobin 9.7 g/dL (13.6–17.5 g/dL) with mean corpuscular volume 86 fL (80–100 fL), and platelets 117/mm** ^**3**^ **(140–450/mm** ^**3**^ **). Peripheral flow cytometry was notable for 1% myeloid blasts but had no evidence of lymphoproliferative disease. The peripheral blood smear was otherwise normal, including no schistocytes. The chemistry panel was notable for a bicarbonate of 16 mmol/L (22–29 mmol/L) and anion gap of 20 (4–14). Total bilirubin was 0.4 mg/dL, AST 103 U/L (5–44 U/L), ALT 117 U/L (10–61 U/L), and alkaline phosphatase 142 U/L (38–108 U/L). Venous blood gas showed a pH of 7.34, pCO2 45 mm Hg, and lactate 10.3 mmol/L. Lactate dehydrogenase (LDH) was 743 U/L (125–243 U/L), erythrocyte sedimentation rate 14 mm/h (0–10 mm/h), and C-reactive protein 8.3 mg/L (< 5.1 mg/L). Serial troponins, international normalized ratio, partial thromboplastin time, fibrinogen, and thyroid function tests were within normal limits. Antinuclear antibody, HIV, and hepatitis B and C serologies were negative.**

This presentation is consistent with a hematologic malignancy. Blasts in the peripheral blood should always prompt additional investigation, including hematology consultation with possible bone marrow aspiration and biopsy. Concurrent anemia and thrombocytopenia suggest decreased bone marrow function. With the elevated LDH, a microangiopathic hemolytic process is possible but less likely given the lack of schistocytes with normal bilirubin and coagulation testing. Since flow cytometry specifically identified myeloid blasts, the highest concern is for a myeloid malignancy.

We should now re-evaluate the previous data. Epistaxis may be related to thrombocytopenia or coagulopathy and can be a presenting sign of leukemia, though his platelets are not low enough to typically cause bleeding. Type B lactic acidosis could be indicative of malignant liver infiltration or of the “Warburg effect,” by which malignant cells generate lactic acidosis via primary utilization of glycolysis regardless of oxygen levels.

Here is my updated PR: 65-year-old man with remote history of gastric cancer presenting with antibiotic-refractory chronic rhinosinusitis, co-localizing symptoms of unilateral hearing loss and epistaxis, and B symptoms, found to have type B lactic acidosis and peripheral blasts, overall concerning for myeloid malignancy.

*As the discussant receives new data, the PR is continually refined to re-focus on the most striking (and typically most diagnostically useful) features. The peripheral blasts and pancytopenia now implicate myeloid malignancy as the most likely diagnosis.*

**The patient underwent computed tomography of the chest, abdomen, and pelvis with and without contrast, which was notable for acute multifocal splenic infarctions, biapical lung scarring with apical pleural calcifications, and an apical calcified granuloma. After 2 L of isotonic fluid, the lactate remained 11 mmol/L. Empiric vancomycin and piperacillin-tazobactam were started; these were discontinued when peripheral blood cultures demonstrated no growth at 24 h.**

Imaging was notable for splenic infarctions, which can be caused by hematologic malignancies, though this finding is often accompanied with splenomegaly. Whenever viscus infarctions are diagnosed, risk factors for bland emboli should be investigated, and septic emboli must be ruled out; negative blood cultures make a typical endovascular infection less likely.

Calcified pulmonary granulomas can represent sequelae of infection such as tuberculosis (TB). The patient has previously lived in China, a country with a high TB burden, and so testing for TB is indicated. San Francisco County has triple the national rate of active TB. In 2022, the rate among Asian/Pacific Islander individuals was 12 times that of non-Hispanic White individuals, with immigrants born in countries with high TB burden comprising the majority of cases.^5^

Here is my updated PR: 65-year-old man with remote history of gastric cancer who immigrated to San Francisco from Guangzhou 13 years ago presenting with symptoms both systemic and co-localizing to the head, found to have type B lactic acidosis, peripheral blasts, splenic infarctions, and a calcified pulmonary granuloma, overall concerning for myeloid malignancy and possible TB.

*The discussant has now selectively incorporated the patient’s corresponding geographic exposures into the PR. Notably, despite the local statistic using race-based categories, the discussant has chosen not to directly incorporate race into the one-line assessment. Instead, he recognizes the mechanism of this disparity is related to exposures based on country of origin and thus frames the risk accordingly. Notice also that the length of the PR has grown despite efforts at compression (“systemic and co-localizing symptoms”). This often typifies ongoing diagnostic uncertainty, as disparate data are not yet able to be reconciled.*

**A bone marrow biopsy revealed 1.1% myeloid blasts and a relative increase in natural killer (NK) cells without definitive evidence of a lymphoproliferative disorder. An interferon-gamma release assay test returned positive. The lactate remained 11–12 mmol/L with pH 7.33–7.36 and normal pCO2.**

The bone marrow biopsy does not reveal acute leukemia, with a blast population well below the diagnostic 20% cutoff based on blast percentage alone (< 5% bone marrow blasts is considered normal or physiologic).

The bone marrow demonstrates a relative increase in NK cells. NK cell large granular lymphocyte leukemias are rare and often associated with peripheral lymphocytosis and large granular lymphocytes on blood smear—neither are present. However, there is a nasal subtype of extranodal NK/T-cell lymphoma, associated with Epstein-Barr virus (EBV), which has higher prevalence in East Asia (including China) and Central/South America.

Nasal NK/T-cell lymphoma usually presents as localized nasopharyngeal disease and involves a destructive mass at the sinuses or palate; diffuse lymphadenopathy is rare. Bone marrow involvement can occur, though this is not a disease hallmark. Additional testing for NK/T-cell lymphoma is warranted.

Of the several unexplained features, vertigo and hearing loss localize to the central nervous system (CNS) and provide a helpful foundation to further revise the differential diagnosis. Intravascular large-cell lymphoma can also present with fever, night sweats, and weight loss, as well as rapidly progressive neurologic signs. Nasopharyngeal carcinoma, another EBV-linked malignancy, could explain epistaxis but rarely causes neurologic deficits and does not explain the peripheral myeloblasts. The patient has at least latent TB; active disseminated TB can cause diverse symptoms including CNS and bone marrow involvement.

Here is my updated PR: 65-year-old man with remote history of gastric cancer who immigrated to San Francisco from Guangzhou 13 years ago presenting with symptoms both systemic and co-localizing to the head, found to have type B lactic acidosis, peripheral blasts, splenic infarctions, increased bone marrow NK cells, and TB infection, overall concerning for NK/T-cell lymphoma (nasal type). My prioritized differential diagnosis is: (1) NK/T-cell lymphoma (nasal type); (2) intravascular lymphoma; (3) myeloid neoplasm; (4) nasopharyngeal carcinoma; (5) disseminated TB.

*With a considerably narrowed differential, the discussant considers how the patient’s geographic exposures (rather than race) impact the probability of specific malignancies. By combining this social history with the symptoms localizing to the head, he now focuses on the nasal subtype of extranodal NK/T-cell lymphoma.*

**Nasal endoscopy revealed a polypoid mass obstructing the left nasal cavity, which was biopsied. Two sputum acid-fast bacillus smears with Mycobacterium tuberculosis polymerase chain reaction (PCR) assays were negative. The lactate increased to 16 mmol/L, with pH 7.27–7.29 and normal pCO2.**

Active pulmonary TB is now significantly less likely, especially without pulmonary symptoms, but we cannot definitively rule it out until cultures are finalized.

The nasal mass likely is malignant. A lymphoreticular malignancy such as NK/T-cell lymphoma (nasal type) remains the strongest possibility. This disease has an increased incidence in East Asia and is diagnosed at higher rates in men compared to women (although most data reports prevalence across sex, there are limited data in non-binary and transgender individuals).^6^ The association with East Asia is thought to be due to environmental exposures, but the sex predilection is not well explained.^7^ Lymphoma is compelling due to several features not readily explained by more common nasal cancers: B symptoms, splenic infarctions, type B lactic acidosis, and elevated LDH. The peripheral myeloid blasts and pancytopenia raise concern for a process involving the bone marrow, but the significance of increased NK cells in the bone marrow is uncertain, as these may be pathologic or reactive. Regardless, NK/T-cell lymphoma most often presents at a localized stage, so the absence of marrow involvement would not rule it out.^8^

While undergoing diagnostic workup for malignancy, it is never too early to start considering a patient’s beliefs about biomedical models for health, values around end-of-life, and planning regarding future access to oncologic care. In the U.S., cancer care access, and thus associated outcomes, remains inequitable. For example, between 2008 and 2017, immigrants to the U.S. from China had higher cancer mortality than U.S.-born Chinese individuals,^9^ which may be due to barriers to diagnosis and/or guideline-concordant treatment. Discussions about treatment planning must be tailored to the individual patient and family rather than relying on stereotypes, assumptions, or prior experiences with ethnically similar patients.

Here is my final PR: 65-year-old man who immigrated to San Francisco from Guangzhou 13 years ago presenting with systemic and co-localizing cranial symptoms, found to have a nasal mass, increased bone marrow NK cells, peripheral blasts, splenic infarctions, and likely latent TB, overall concerning for NK/T-cell lymphoma (nasal type) with marrow involvement, complicated by type B lactic acidosis.

*In the final PR, the discussant has continued to include a specific geographic exposure (immigration from Guangzhou) that plausibly contains a pathophysiologic link to the most likely diagnosis (NK/T-cell lymphoma (nasal type)). The discussant has also removed gastric cancer, as the workup narrowed the differential diagnosis to unrelated conditions. While prior cancer is often relevant when contextualizing an undifferentiated finding, once it becomes clear the diagnosis is unassociated, this past history is unnecessary as the new disorder is framed.*

**Sinus MRI revealed a large solid mass with subcutaneous, perineural, and intracranial spread (**Fig. 1**). Serum EBV IgG and PCR were positive. Pathology confirmed NK/T-cell lymphoma (nasal type). Additional bone marrow staining also revealed extranodal NK/T-cell lymphoma (40% of marrow cellularity). He began urgent chemotherapy, and the lactate normalized within 4 days. His final diagnoses were type B lactic acidosis due to the Warburg effect from stage IV extranodal NK/T-cell lymphoma (nasal type), as well as latent TB. Unfortunately, 3 months later he developed severe, refractory lactic acidosis during a hospitalization for chemotherapy and died.**

**Figure 1:**
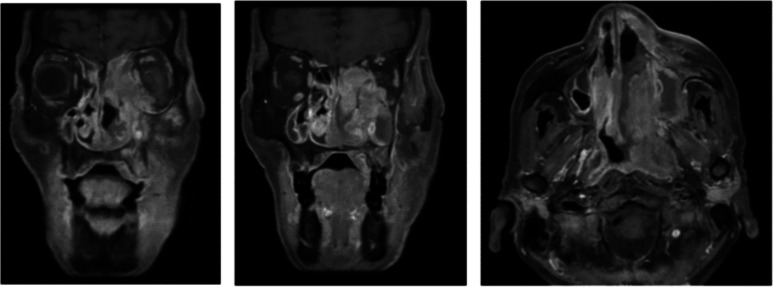
Sinus MRI. This MRI showed a large, solid mass centered in the left nasal cavity with extensive local and multicompartmental invasion, as well as subcutaneous, perineural, orbital, and intracranial spread.

## DISCUSSION

In this case, an older man was admitted with severe type B lactic acidosis, which was eventually diagnosed as the Warburg effect secondary to stage IV NK/T-cell lymphoma. This case showcases the differential diagnosis for type B lactic acidosis and also highlights the need to be thoughtful when considering the patient’s racial identity and geographic exposures in clinical reasoning.

The magnitude of the presenting lactic acidosis made it the focal point of the initial PR. The discordance between its severity and the patient’s clinical stability rapidly confirmed type B lactic acidosis, and the discussant was able to systematically narrow the differential diagnosis. While the potential causes of lactic acidosis are broad, typically only cancer or thiamine deficiency can cause such dramatic type B elevations. Contextualization of the laboratory result facilitated rapid recognition of the Warburg effect.

We believe the routine inclusion of social context in a PR should be avoided when the differential is broad (e.g., early in the reasoning process), as its use could lead to inappropriate anchoring—and its influence on the differential should be invoked only when there is a well-described mechanism between a component of the social context and a specific diagnosis. To avoid falsely conflating race with biology and pathology, race, in particular, should only be referenced when experiences of interpersonal structural racism are directly related to the presentation. In short, we advocate for race-conscious, as opposed to race-based, clinical reasoning.^10^

For example, the patient was born in and lived most of his life in Southeast China, a region with epidemiologic links to NK/T-cell lymphomas. However, to properly use this information in our diagnostic reasoning while minimizing implicit bias and premature closure, we needed to understand the possible mechanisms of these associations and to judiciously apply our knowledge of his specific social history. The mechanism explaining higher prevalence of NK/T-cell lymphomas in China is not fully understood, but EBV infection likely plays a role. Extranodal NK/T-cell lymphomas are universally associated with EBV infection,^6^ and it is possible that certain EBV subtypes are specifically associated.^11^ Other EBV-associated malignancies also have well-described higher prevalence in certain areas of Asia due at least in part to environmental exposures.^12^ For EBV-associated nasopharyngeal carcinoma, there is a decrease in prevalence in second-generation Chinese individuals living in the U.S., further implying key environmental factors.^12^ Given this important relationship between pathophysiology and geography, along with its diagnostic value, the selective incorporation of the patient’s geographic history in the later stages of the PR is justified.

### Clinical Teaching Points

- Type B lactic acidosis can be due to malignancy, thiamine deficiency, medications, liver or kidney disease, alcohol use, HIV infection, or congenital causes.
- Hospitalists should maintain a high clinical suspicion for type B lactic acidosis for an occult hematologic malignancy when it is severe and unexplained.
- Nasal NK/T-cell lymphoma is a malignancy that is especially prevalent in China, associated with EBV infection, and found more frequently in men.
- Being specific about a patient’s social history, as well as understanding potential mechanisms that may link a disease with a patient’s identity, can enhance evidence-based clinical reasoning and counter the potential biases caused by using poorly defined racial or geographic identifiers in the PR.
